# Neural Network-Based Optimization of an Acousto Microfluidic System for Submicron Bioparticle Separation

**DOI:** 10.3389/fbioe.2022.878398

**Published:** 2022-04-19

**Authors:** Bahram Talebjedi, Mohammadamin Heydari, Erfan Taatizadeh, Nishat Tasnim, Isaac T. S. Li, Mina Hoorfar

**Affiliations:** ^1^ School of Engineering, University of British Columbia, Kelowna, BC, Canada; ^2^ Department of Chemistry, The University of British Columbia, Kelowna, BC, Canada; ^3^ Faculty of Engineering and Computer Science, University of Victoria, Victoria, BC, Canada

**Keywords:** acoustofluidics, microfluidics, particle separation, artificial neural network, multi-objective optimization, pareto front

## Abstract

The advancement in microfluidics has provided an excellent opportunity for shifting from conventional sub-micron-sized isolation and purification methods to more robust and cost-effective lab-on-chip platforms. The acoustic-driven separation approach applies differential forces acting on target particles, guiding them towards different paths in a label-free and biocompatible manner. The main challenges in designing the acoustofluidic-based isolation platforms are minimizing the reflected radio frequency signal power to achieve the highest acoustic radiation force acting on micro/nano-sized particles and tuning the bandwidth of the acoustic resonator in an acceptable range for efficient size-based binning of particles. Due to the complexity of the physics involved in acoustic-based separations, the current existing lack in performance predictive understanding makes designing these miniature systems iterative and resource-intensive. This study introduces a unique approach for design automation of acoustofluidic devices by integrating the machine learning and multi-objective heuristic optimization approaches. First, a neural network-based prediction platform was developed to predict the resonator’s frequency response according to different geometrical configurations of interdigitated transducers In the next step, the multi-objective optimization approach was executed for extracting the optimum design features for maximum possible device performance according to decision-maker criteria. The results show that the proposed methodology can significantly improve the fine-tuned IDT designs with minimum power loss and maximum working frequency range. The examination of the power loss and bandwidth on the alternation and distribution of the acoustic pressure inside the microfluidic channel was carried out by conducting a 3D finite element-based simulation. The proposed methodology improves the performance of the acoustic transducer by overcoming the constraints related to bandwidth operation, the magnitude of acoustic radiation force on particles, and the distribution of pressure acoustic inside the microchannel.

## 1 Introduction

Contactless manipulation and conversion of biological sample mixtures to distinct populations is a critical step in many bioanalytical and biomedical applications such as water quality assessment (Carugo et al., 2014), disease diagnosis and prognosis (Talebjedi et al., 2021a), (Antfolk and Laurell, 2017), and public health monitoring (Li et al., 2017). The majority of traditional particle separation techniques commonly used in clinical settings require intricate sample preparation, bulky equipment, and a high volume of samples (Talebjedi et al., 2021a). Conventional label-free methods are density gradient-based techniques involving centrifugation; however, this method has multiple drawbacks, such as low bioparticles’ recovery rate, viability, and functionality (Yang et al., 2002). The microfluidics platform offers an accurate and sensitive sorting of different types and sizes of bioparticles by decreasing the reaction time, consumed reagents, manufacturing cost, and experimental time (Zhang et al., 2016). There have been different microfluidics particle enrichment methods such as on-chip centrifugation (Yeo et al., 2018), deterministic lateral displacement (DLD) (Wunsch et al., 2016), filtration (Liang et al., 2017), viscoelastic flow (Liu et al., 2016), inertial focusing (Li et al., 2021) and acoustofluidic (Ramshani et al., 2019), (Dalili et al., 2019). Among these techniques, acoustic-based microfluidic devices have attracted extensive attention due to wide-ranging advantages in throughput, biocompatibility, and compact device size (Xie et al., 2020). Moreover, the separation is label-free and only relies on biophysical markers such as density and compressibility (Ahmed et al., 2018). The microfluidics acoustic-based devices benefit from acoustic radiation force for micro/nanoparticle confinement at predefined locations in a continuous flow. There are two main acoustic-based separation techniques, bulk acoustic wave (BAW) based systems (Leibacher et al., 2015), and surface acoustic wave (SAW) based systems (Wang et al., 2019a), (Fu et al., 2017). In SAW-based microdevices, a pair of interdigitated transducers electrodes are patterned at two sides of the fluidic channel, generating two opposite-direction surface acoustic waves by the piezoelectric surface actuation. The constructive and destructive interference of two surface acoustic waves forms standing surface acoustic waves (SSAW) and radiates acoustic energy into the flow domain. The formation of SSAW results in the periodic distribution of pressure nodes (PNs) and antinodes (ANs) inside the microchannel. The particles inside the microchannels migrate laterally in response to acoustic radiation force and will be pushed towards the minimum or maximum acoustic radiation pressure lines based on their acoustic contrast factor (Taatizadeh et al., 2021). The particles with positive acoustic contrast factor (e.g., cells and vesicles suspended in aqueous solutions) are pushed towards the pressure nodes and particles with negative acoustic contrast factor (e.g., some subgroups of lipoproteins) are pushed to the pressure anti-nodes by the acoustic radiation force. As the acoustic radiation force is proportional to the volume of the particles, the amplitude of the applied acoustic radiation force due to the size difference of the suspended particles results in different lateral migration across the channel and makes the separation possible.

There have been several studies in the manipulation and separation of submicron bioparticles. A study conducted by Lee et al. (2015), presented an ultrasound transducer for size-specifically separation of Microvesicles (MVs) with high separation yield and resolution in a continuous and contact-free manner. They showed that their acoustic filtering technique is fast, gentle on vesicles, and compatible with limited sample volumes compared to conventional isolation methods. Another study conducted by Wang et al. (2020), introduced an acoustofluidic platform for size-based isolation of exosomes from a saliva sample. Compared to conventional isolation methods (e.g., gold standard, differential centrifugation, droplet digital RT-PCR analysis), it was seen that the average yield for the suggested acoustic-based device was 15 times higher. Zhao et al. (2020) developed a disposable acoustofluidic platform with unidirectional interdigital transducers for nano/microparticle separation. The results indicated that the exposed acoustic radiation force to the particles with the disposable channel was comparable with the permanently bonded devices. The suggested unidirectional IDT-based device could differentiate bacteria from human red blood cells (RBCs) with 96% purity. In a study conducted by Liu et al. (2021), the authors developed a microfluidics platform for separating the blood cells from plasma by using acoustic microstreaming. The blood plasma separation was performed with 31.8% plasma yield and 99.9% plasma purity.

Many parameters such as microchannel and IDT geometry, working voltage, flowrate, etc., are involved in defining the performance efficiency of the acoustofluidic devices that need to be tuned and optimized. Two main critical parameters that drastically influence the performance and cover both electrical and mechanical characteristics of the acoustofluidic devices are the reflection coefficient and bandwidth. The acoustofluidic devices should be designed to show the minimum reflection coefficient and maximum bandwidth for effective isolation of particles from a wide size range (from sub-micron to micron) without significant heat and bubble generation. As the acoustofluidic devices work under radio frequency signals (RF), the RF generator and transmission line impedance should be matched with load for the highest power delivery to the load. Any impedance mismatch between the source and load causes the signal to be reflected to the source (Wang et al., 2019b). The best way to address the impedance matching problem is designing the impedance of the acoustofluidic device equal to the source impedance (function generator), which is usually 50 Ω . More information regarding the impedance matching can be found in the SI document. However, due to the lack of accurate analytical solutions for correlating the geometrical features of the acoustic device with its corresponding impedance, statistical approaches (such as machine learning) can be implemented for laying-out performance prediction platforms with high accuracy (Talebjedi et al., 2021b). The machine learning approach has also been implemented to optimize and automate acoustic-based separation techniques. However, the machine learning techniques have not been extensively applied to this field, and it is only limited to a handful number of research articles. In a study conducted by Yiannacou and Sariola (2021), a machine-vision based particle position measurement combined with machine learning approach was proposed by frequency controlling of a single acoustic transducer. They demonstrated that their methodology is easy to implement and adaptable to different chip designs, fluid properties and particle sizes. The other study utilizing the machine learning method conducted by Turan et al. (2018), proposes a machine learning-based optimization of a pillar-based microchip for T-cells and B-cells isolation and detection. The micropillar array was optimized for trapping leukocytes while letting the remaining blood cells flow through.

In this study, we leverage a robust machine learning-based framework for performance prediction and optimization of an acoustic transducer for a diverse set of IDTs geometrical configurations. The multi-layer perceptron (MLP) neural network was developed to create an accurate radio frequency prediction platform for acoustofluidic devices. As the geometrical features of the IDTs are the main influential parameters on the radio frequency response of the SAW resonator, the electrodes’ length, distance and numbers are considered as design parameters, and reflection coefficient and quality factors were taken as output parameters. After developing the neural networks and performing the accuracy assessments, two developed neural networks were fed to a multi-objective genetic algorithm (MOGA) to be optimized for minimum reflection coefficient and quality factor measures by establishing a Pareto-optimal front. The sensitivity analysis on reflection coefficient and bandwidth variation was carried out by conducting different experimental and numerical studies. The result of this study shows that optimization of the power loss and bandwidth of the acoustofluidic device has a remarkable influence on the amplitude of the acoustic radiation force and the pattern of the acoustic pressure distribution inside the microchannel.

## 2 Material and Method

### 2.1 Fabrication Process

Due to perfect optical transparency and high electromechanical coupling coefficient of lithium niobate (LiNbO_3_) over other piezoelectric substrates, A 128° YX cut LiNbO_3_ was used for SAW generation. The acoustic microdevice consisted of a LiNbO_3_ substrate with gold interdigitated electrodes patterned on the surface and a polydimethylsiloxane (PDMS) microchannel bonded between the area of two IDTs. For fabricating the IDTs on the substrate, first, a 200 Å of chromium layer followed by 1,000 Å of the gold layer was sputtered on double-side polished 128° YX LiNbO_3_ substrate by sputtering machine (Angstrom Engineering Inc.). In the next step, the wafer was spin-coated with an S1813 positive photoresist (MicroChem Corp.) to form a 1μm photoresist film on the wafer surface. The electrode patterning was followed by wafer exposure to the UV light using a mask aligner (Model 200, OAI). The wet etching process first started by removing the unwanted photoresist using MF-319 developing solution (MicroChem Corp.). The wafer was then rinsed with chromium and gold etchant to remove the undesirable metal sputtered regions. Finally, the thin photoresist film on top of the electrodes was removed with an 1,165 photoresist stripper. The microchannel fabrication was performed by a standard soft lithography process using a negative SU8-2025 photoresist (MicroChem Corp.). The fabricated PDMS microchannel was bonded on the LiNbO_3_ substrate through oxygen plasma treatment. The oxygen plasma machine was set for 25 seconds , 25 W , 12 sccm oxygen flowrate and 170 mTorr of pressure.

### 2.2 Experimental Setup

**FIGURE 1:**
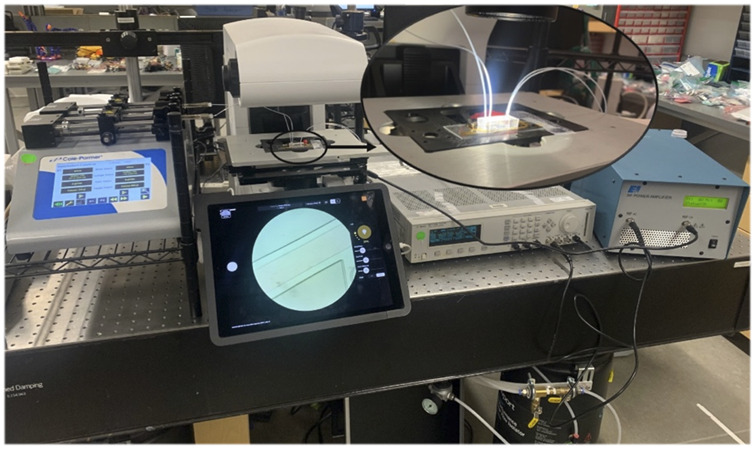
A view of experimental setup including syringe pump, microscope, function generator, and RF amplifier.

The experimental setup comprised of acoustofluidic microchip mounted on an inverted fluorescent microscope (REVOLVE 4, ECHO). The SSAW was formed by applying a sinusoidal signal to the IDTs. The function generator (81110A, Agilent Technologies Inc.) was employed for providing the RF signal, and the output of the function generator was connected to a power amplifier (325LA, Electronics & Innovation Ltd.) for amplifying the transmitted power. The process of monitoring the power amplification and wave formation was done by an oscilloscope (DSOX 2024A, Keysight). Two individual syringe pumps (Cole-Parmer Instrument Company LLC.) controlled the sample and sheath-flow flowrate. The PTFE microtubes (Fisher Scientific) with an inner diameter of 0.413 mm were used to connect microchannel inlets with glass syringes. The VNA machine (E5061A, Agilent) was used to obtain each piezo actuator’s frequency response. Figure 1 demonstrates the experimental setup designated for running experimental tests.

### 2.3 Neural Network Design

The Artificial Neural Networks (ANNs) capability in highly nonlinear data recognition in multivariate systems makes them an ideal technique for microfluidics design automation applications (Rizkin et al., 2019; Honrado et al., 2020; Lashkaripour et al., 2021). In most of the microfluidic devices, there are some dominating parameters (such as flowrates, voltage, geometrical features etc.) that significantly influence the performance of the micro devices; tuning these parameters for the desired application is one of the major challenges of the field. The prediction capability of the machine learning platforms makes them an ideal tool for eliminating trial and error in the design process. This technique could be easily applied for design automation of different types of active and passive microfluidics devices (such as micromixers, dielectrophoresis, and optical microseparators). In this paper, the feedforward backpropagation neural network based on Levenberg-Marquardt algorithm is proposed for the quality assessment of acoustofluidic devices. Levenberg-Marquardt is a supervised technique for properly tuning the neural network weights, which fine-tunes the weights according to the gradient of a loss function calculated in the previous epoch (i.e., iteration). This method is an adaptive function network that utilizes the Jacobian matrix and evaluates the network performance as the mean squared of the error. The MSE measure between the desired (d) and target (t) output value is shown in Eq. 1.

$$E\left(n\right)=\frac{1}{2}\sum_{i=1}^{m}{\left(t_{i}\left(n\right)-d_{i}\left(n\right)\right)}^{2}$$ (1)

where n and m are training epochs and the number of outputs, respectively, the general formulation for computing the output in each hidden layer and the output layer has been expressed in Eq. 2 and Eq. 3.

W(n+1)=W(n)+ΔW(n) (2)

$$\text{Δ}W\left(n\right)=-\eta \frac{\partial E\left(n\right)}{\partial W\left(n\right)}=-\eta \nabla E\left(n\right)$$ (3)

where η is the learning rate. The learning rate hyperparameter range is usually between 0.01 and 0.1. The alteration of the learning rate value controls the training speed and the amount of allocated error in updating the weights. In this method, the Hessian matrix and gradient of performance index are represented as Eq. 4 and Eq. *5* respectively.

$$H\left(n\right)=J^{T}\left(n\right)J\left(n\right)$$ (4)

$$g\left(n\right)=J^{T}\left(n\right)E\left(n\right)$$ (5)

where term J is the Jacobian matrix containing the first derivatives of the network errors with respect to the weights and biases, and term E is the network error vector. The Levenberg-Marquardt algorithm minimizes the error function by keeping the step size small to certify the validity of the linear approximation. By utilizing the Hessian matrix approximation and minimizing the modified error, the Newton-like update equation is obtained as Eq. 6.

$$w\left(n+1\right)=w\left(n\right)-{\left[J{\left(n\right)}^{T}J\left(n\right)+\mu I\right]}^{-1}J{\left(n\right)}^{T}E\left(n\right)$$ (6)

where μ is the nonnegative step size governing parameter and I is the identity matrix. For μ=0 , the Eq. 6 is Newton’s method; however, for large measures of μ, the equation is changed to gradient descendent with a small step size. In gradient descendent optimization technique, the learning rate must be adjusted to an appropriate value (neither too low nor too high) to avoid bouncing back and forth between gradient descent function.

### 2.4 Multi-Objective Optimization

**FIGURE 2:**
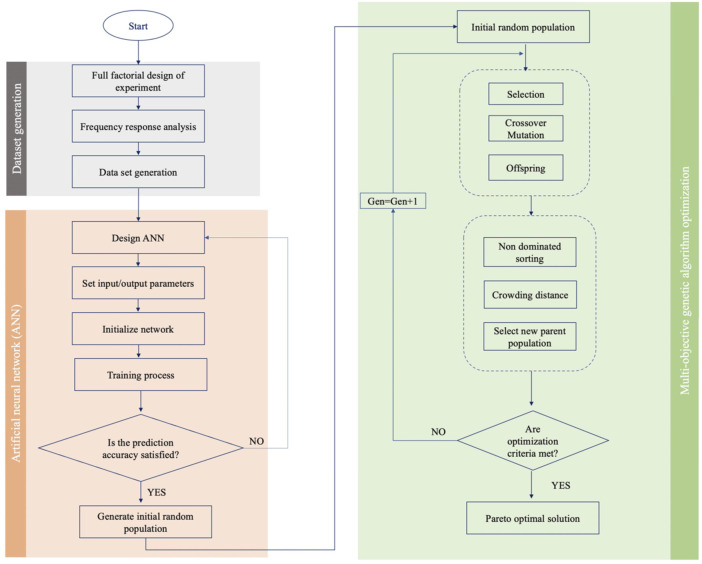
Flowchart of ANN-GA algorithm.

Combining neural networks with optimization algorithms can provide a robust optimization platform for microfluidic systems. The multi-objective heuristic optimization approaches have shown their superiority in tuning the operating parameters and design optimization of microfluidic devices (Talebjedi et al., 2021c). In this study, for identifying the optimum combination of the design factors for minimum reflection coefficient and quality factor, the trained neural network model was used in connection with NSGA-II multi-objective genetic algorithm. The GA first starts by generating an initial population of the chromosome, which are a possible solution for genetic algorithm optimization. The fitness function is used for determining the best chromosomes with highest survival ability and reproducibility. Here, the fitness function was created according to the developed ANN model. Individuals’ competition with their parents produces the offspring for next-generation creation, so that the superb individuals are retained, leading to a rise in the overall population evolution level. The newly generated population outperforms the previous population by continuing the process of selection, crossover and mutation on each generation (Talebjedi et al., 2020). Here, the Nondominated Sorting Genetic Algorithm (NSGA-II) is utilized for global optimization objectives of the reflection coefficient and quality factor as the algorithm is fast and avoids local optimal solution. The multi-objective optimization problems return the Pareto-optimal solution, showing a trade-off between objective functions subjected to defined constraints. The suggested solutions on the Pareto front are equally suitable and none of them have superiority over others. The decision-maker chooses between points according to the application to meet the required criteria. The flowchart of the ANN-GA algorithm is shown in Figure 2.

### 2.5 Governing Equations

The computation of harmonic acoustic pressure distribution was obtained by Helmholtz wave equation defined as follow:

$$\nabla .\left(-\frac{1}{{\bar{\rho }}_{k}}\nabla P\right)-\frac{\omega^{2}}{{\bar{\rho }}_{k}{\bar{c}}_{k}^{2}}P=0,$$ (7)

where P  is the acoustic pressure and ${\bar{\rho }}_{k},{\bar{c}}_{k}$ and ω are the equivalent density of the medium, equivalent sound velocity of the medium and angular frequency. The fluid flow simulation was performed by solving the continuity and momentum equations as follow:

$$\frac{\partial \rho }{\partial t}+\nabla .\left(\rho u\right)=0,$$ (8)

$$\rho \left(\frac{\partial u}{\partial t}+u.\nabla u\right)=-\nabla P+\mu \nabla^{2}u+\left(\mu_{b}+\frac{1}{3}\mu \right).\text{u}$$ (9)

where ρ is the density of the fluid, P is the pressure, u is the fluid flow velocity, $\mu_{b}$ is the bulk fluid viscosity. The linear piezoelectric constitutive equations are shown as follow:

$$\sigma_{ij}=C_{ijkl}s_{kl}-e_{ijm}E_{m},$$ (10)

$$D_{m}=e_{mij}s_{ij}+\varepsilon_{mk}E_{k},$$ (11)

where σ and D  are mechanical stress tensor and electric displacement vector, respectively. The term E shows the electric field, $C_{ijkl}$ is the fourth-order elasticity matrix, tensor, $\varepsilon_{mk}$ is the second-order tensor of the dielectric matrix, $s_{kl}$ is the second-order strain tensor and $e_{ijm}$ is the third-order piezoelectric stress matrix responsible for coupling the electric field with mechanical vibration. The model parameters used for the SAW simulation is listed in Supplementary Table S1.

## 3 Results and Discussion

### 3.1 Validation

**FIGURE 3:**
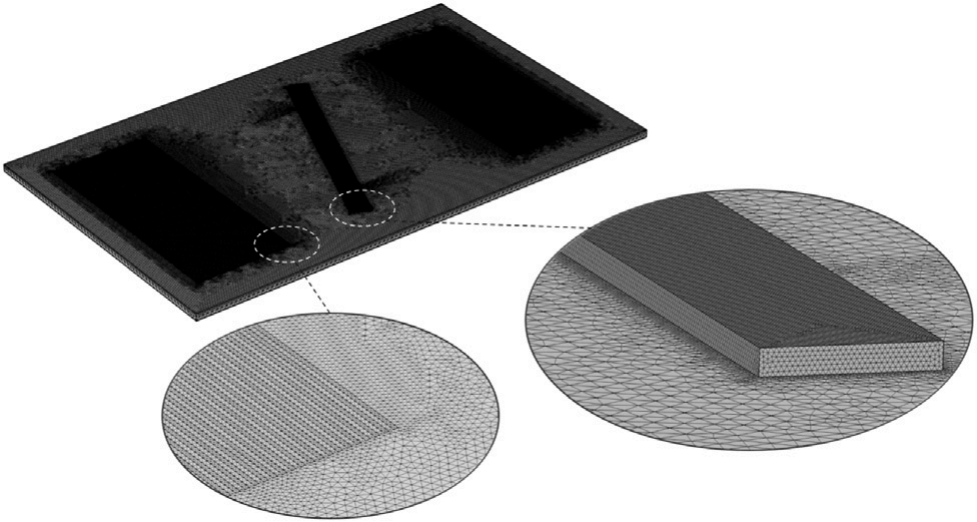
Numerical grids of a 3D acoustic actuator.

**FIGURE 4:**
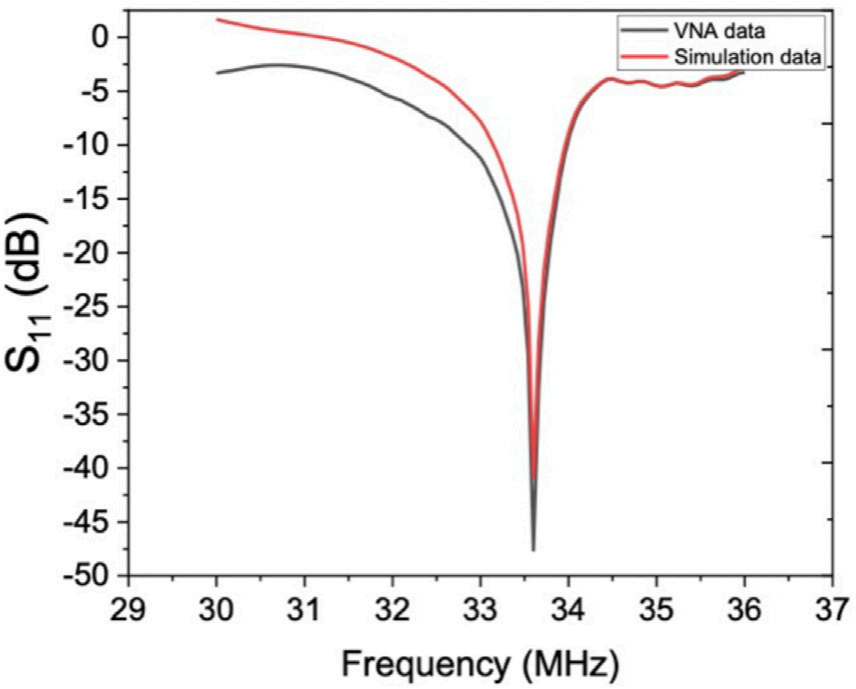
Comparison of the frequency response results from numerical simulation and experimental data for experiment number 108.

In this study, the numerical analysis was performed using the finite element analysis (FEA) software package (COMSOL Multiphysics, version 5.6). The preliminary mesh dependency test was carried out to find the optimum grid size. The resonant frequency and reflection coefficient measures were the controlling parameters for monitoring the convergency test and validation. Four different mesh setups with 754,321, 1,256,798, 1,794,532 and 2,1457,68 grids were used for experiment number 108 simulation. This experiment’s resonant frequency and reflection coefficient are 33.6 MHz and −48 (dB) , respectively. Table 1 represents the resonant frequency and reflection coefficient for different grid setup (coarse to high grid resolution). Table 1 reveals the corresponding discrepancy of the predictions reduced to 2.35% for the resonant frequency and 4.5% for the reflection coefficient for mesh systems based on 1,794,532 and 2,145,768 grids, showing a good converged solution. As a result, the meshing setup with 2,145,768 grid elements was considered for domain discretization. Figure 3 demonstrates the final meshing system of the acoustic actuator and the microchannel. It was seen that the suggested grid setup from the grid dependency test shows an excellent agreement with the experimental measurements, as illustrated in Figure 4.

**TABLE 1:** Different meshing systems for the Grid-dependency test.

Number of elements	Resonance frequency (MHz)	$S_{11\;}\left(dB\right)$ Magnitude
754,321	37.83	−27
1,256,798	35.72	−33
1,794,532	34.34	−44
2,145,768	33.54	−46

### 3.2 Frequency Response Prediction

**FIGURE 5:**
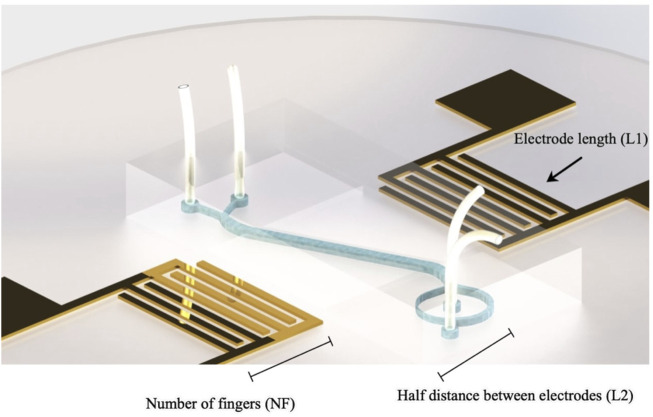
Schematic view of the acoustofluidic microseparator and IDT features.

Here we develop two multi-layer perceptron (MLP) neural networks for creating accurate prediction models of the resonator frequency response (reflection coefficient and Q-factor) according to different IDT geometry features. The dataset was obtained by laying out a full factorial design of experiment (DOE). Three main parameters, including length of the electrodes $\left(L_{1}\right)$ , the half distance between electrodes $\left(L_{2}\right)$ and the number of fingers (NF) were considered as the IDT design parameters with five levels. The schematic of the acoustofluidic device and corresponding IDT features have been demonstrated in Figure 5. The design parameters and their levels have been demonstrated in Table 2. The full factorial experiments (considering all the possible combinations of levels and parameters) were carried out to lay out the database for neural networks. The results of the created design of experiment is reported in Supplementary Table S2. After performing the full factorial design of the experiment, the reflection coefficient and Q-factor measures were extracted and recorded by sweeping the frequency over a wide range of frequencies with a VNA machine. To ensure that the results from each experiment are accurate and reliable, each device was fabricated three times, and the average response was taken as the outcome. After extracting the performance operation features of the conducted experiments, two different neural networks were developed for each objective. The model with the highest accuracy was picked up and saved for prediction purposes. The dataset was split into 15% for validation, 15% for tets, and the rest for training. The prediction models’ performance was assessed by evaluating the coefficient of determination, root mean square error, and mean absolute percentage error. The coefficient of determination usually referred to as the “goodness of fit,” is a value between 0 and 1 that defines the extent of dependent variable variation that the prediction model can explain. The higher value of the determination coefficient reveals that the model is reliable for future forecasting. The corresponding formulas for calculating the coefficient of determination, root mean square error and mean absolute percentage error have been demonstrated in Eq. 12–14 respectively.

$$R^{2}=\frac{{\left[\sum_{i=1}^{n}\left(x_{i}-\bar{x}\right)\left(y_{i}-\bar{y}\right)\right]}^{2}}{\left[\sum_{i=1}^{n}{\left(x_{i}-\bar{x}\right)}^{2}{\left(y_{i}-\bar{y}\right)}^{2}\right]}$$ (12)

$$RMSE=\sqrt{\frac{1}{n}\sum_{i=1}^{n}{\left(x_{i}-y_{i}\right)}^{2}}$$ (13)

$$MAPE=\frac{1}{n}\sum_{i=1}^{n}\frac{\left|x_{i}-y_{i}\right|}{x_{i}}$$ (14)

**TABLE 2:** Microseperator design parameters and levels.

Parameter/Levels	$L_{1}\left(mm\right)$	$L_{2}\left(mm\right)$	NF
1	6	4	20
2	7	5	24
3	8	6	30
4	9	7	34
5	10	8	40

**FIGURE 6:**
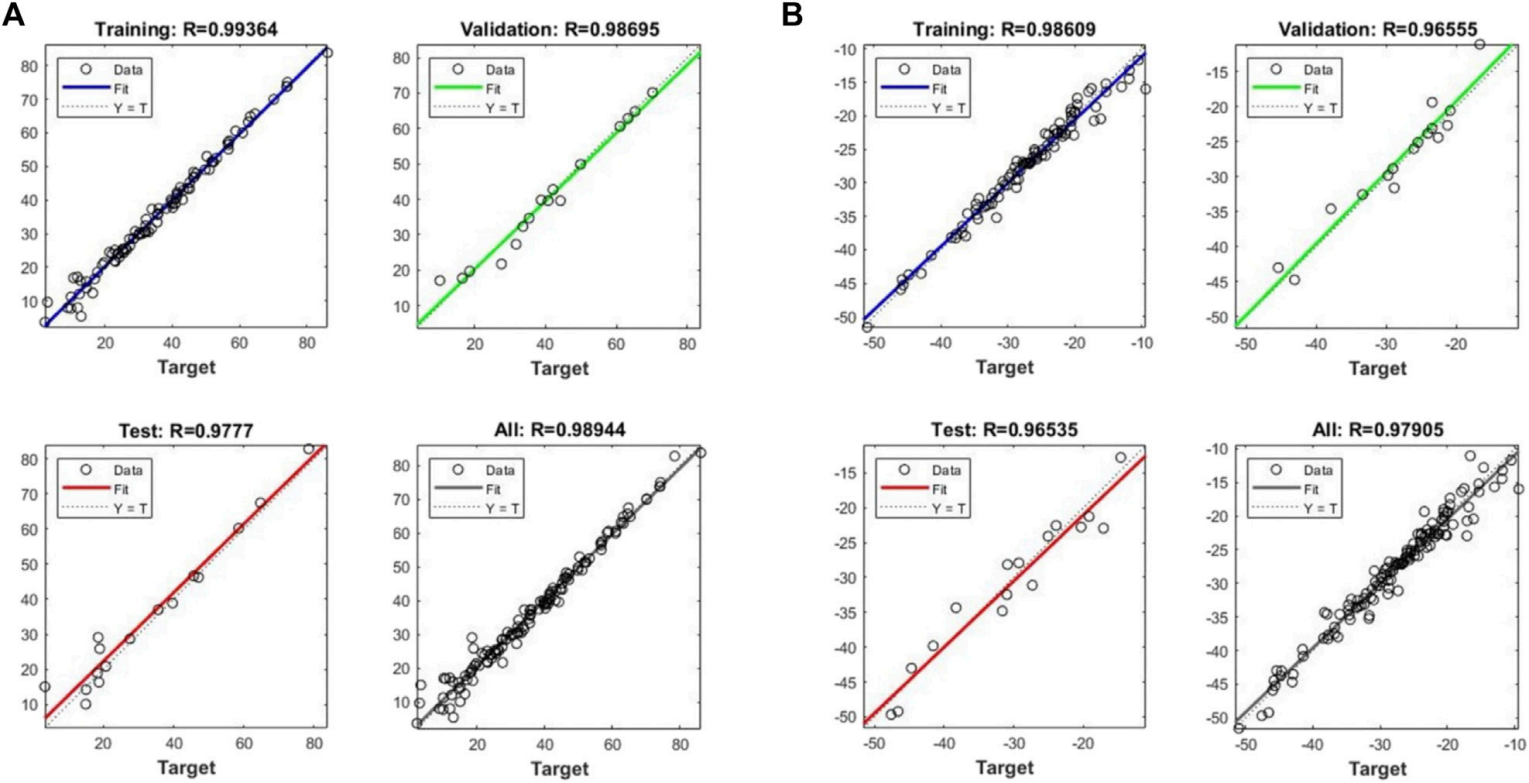
Measures of the R-squared for train, validation, and entire data for **(A)** Reflection coefficient and **(B)** Q-factor models.

**FIGURE 7:**
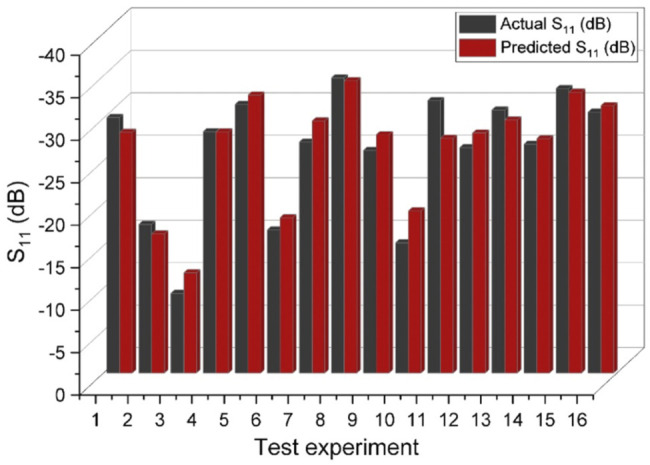
The comparison of actual and predicted reflection coefficient values for different experiments.

**FIGURE 8:**
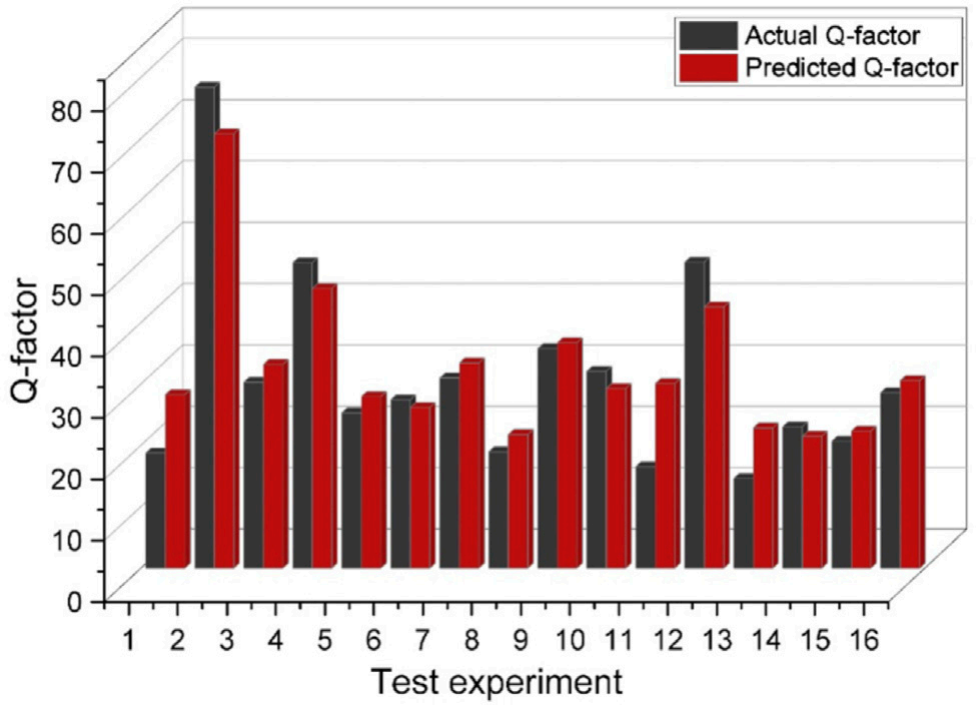
The comparison of actual and predicted Q-factor values for different experiments.

Figure 6 demonstrates the coefficient of determination $\left(R^{2}\right)$ value for training, validation, test, and all the datapoints for reflection coefficient and Q-factor models. As Figure 6 reveals, the coefficient of determination for test data is higher than 0.96 for both networks meaning that the model is not overfitted or under fitted. Table 3 also demosntrates the measures of the coefficient of determination (unseen data), root mean square error (RMSE), and mean absolute percentage error (MAPE) for both artificial neural networks. The RMSE and MAPE measures for refelcetion coefficient model were obtained as 1.044 and 3.23% , respectively, and for the Q-factor network were calculated as 4.75 and 17.82% , respectively. Considering the performance analysis measures, it can be concluded that constructed neural networks are reliable enough to provide an accurate prediction over reflection coefficient and Q-factor. To visualize the prediction ability of the ANNs for completely unseen data, we plotted the predicted and actual values of the test data for both reflection coefficient and Q-factor networks as represented in Figures 7, 8, respectively. Concerning the networks accuracy assessment, it can be concluded that the neural networks can predict the reflection coefficient and Q-factor for unseen data by the algorithm with high accuracy; therefore, the machine learning-based prediction platforms were automated to predict the frequency response of a given IDT design by cutting the trial and error efforts.

**TABLE 3:** The measures of the performance analysis of the prediction model.

	$R^{2}$	RMSE	MAPE
Reflection coefficient	0.97	1.044	3.23%
Quality factor	0.96	4.75	17.82%

### 3.3 Multi-Objective Optimization of Artificial Neural Networks

**FIGURE 9:**
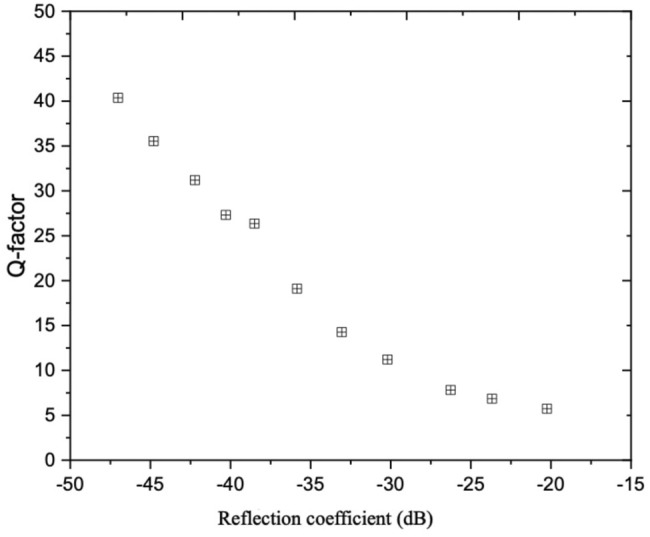
Pareto front of the reflection coefficient and Q-factor models.

To demonstrate the capability of the prediction models in delivering the best optimized user-specified performance in terms of reflection coefficient and Q-factor, we benefited from a heuristic multi-objective optimization approach. In this regard, we considered two ANNs as objective functions of the Genetic algorithm subjected to some constraints. In this study, the constraints were considered as the lower and upper bounds of the IDT features. A Pareto-optimal front was created with an optimized trade-off between the objectives (i.e., minimum reflection coefficient and Q-factor). As each point of the Pareto front is a globally optimal solution, non of the Pareto-optimal solutions have superiority over others for both objectives; thus, the solution’s choice depends on the performance that meets the designer’s need. Figure 9 demonstrates the Pareto front of two objective functions. To examine how multi-objective optimization aids in improving the performance of the acoustofluidic device in separating and confining the particles, we picked up a point on the Pareto front with reflection coefficient as −45 dB and Q-factor as 45 and fabricated the corresponding device. Next, we tested the acoustofluidic device capability in deviating and confining the 0.5 μm polystyrene particles from their initial stream to the upper side of the channel. Supplementary Video S1 shows the confinement of the 0.5 μm polystyrene particles before and after applying the radiofrequency. In this experiment, the frequency was set as 33.7 MHz , voltage as 17 V and inlet flowrates were 1 μl/min for the main particle stream and 4 μl/min for the sheath flow. To compare the functionality of the device setup on the Pareto front and out of the Pareto front, we conducted another experiment with a device reflection coefficient of −12 dB . Supplementary Video S2 shows the device’s performance before and after acoustic excitation at different voltages. As Supplementary Video S2 shows, by applying the RF voltage, the particles focus in parallel lines with the microchannel wall, showing that the acoustic radiation force is not sufficient enough to push the particles to the other side of the channel. Moreover, it could be concluded that the nodal pressure lines are formed parallel to the channel and have not kept their slanted shape. By rising the voltage, the concentration of the particles moving to the middle of the channel increases; however, part of the particles are still located in parallel lines to the channel wall. For voltage 24 V , the more significant portion of the particles get concentrated in the channel center, but we can see that the bubbles start forming in the channel wall due to the acoustic cavitation.

### 3.4 Sensitivity Analysis

#### 3.4.1 Reflection Coefficient

**FIGURE 10:**
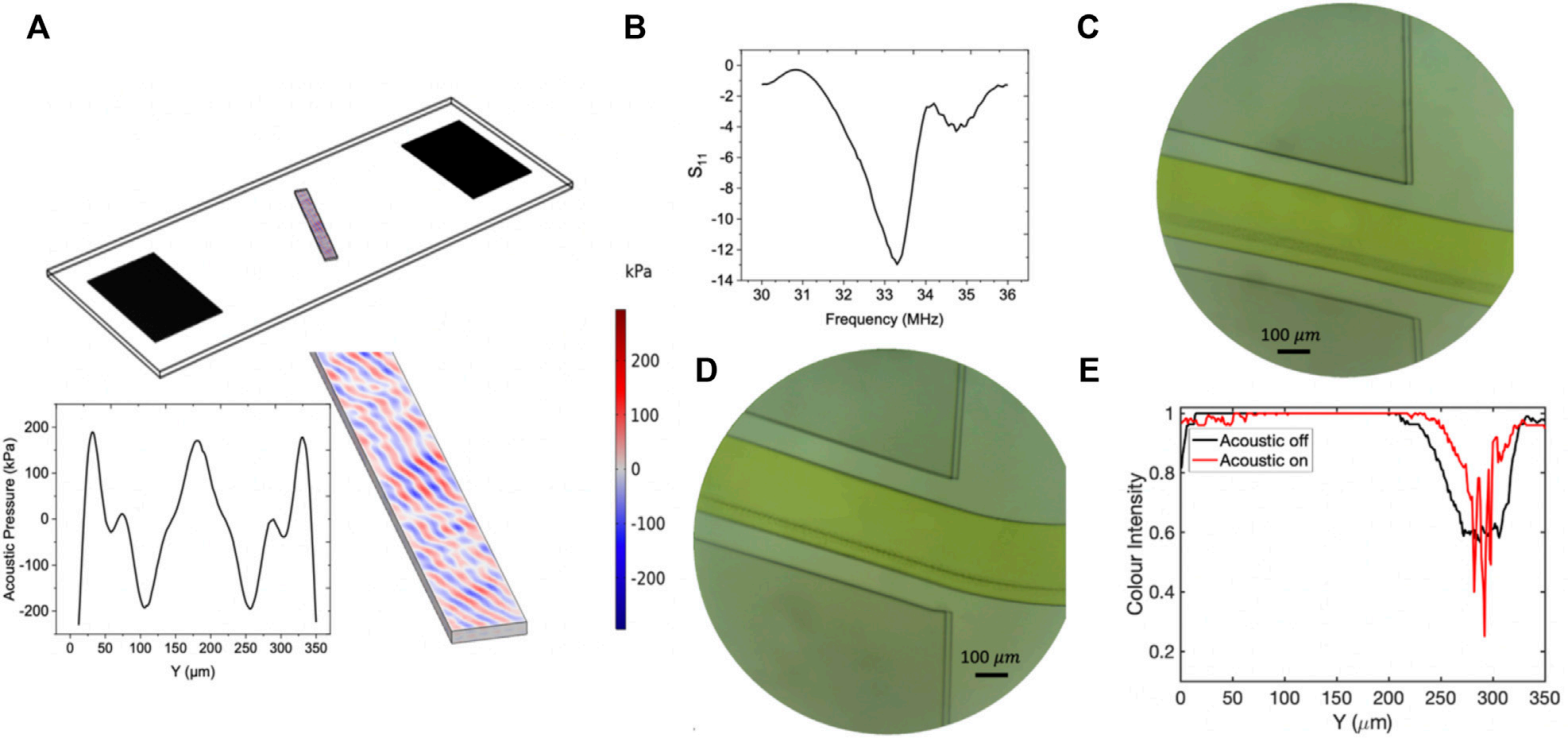
The results of the numerical and experimental studies corresponding to experiment number 36 with tilt angle 20°. **(A)** Contours of the acoustic pressure field distribution inside the fluidic domain. **(B)** The frequency response of the resonator. **(C)** The flow of 0.5 μm particles before applying the acoustic force at the channel’s end. **(D)** Confinement of 0.5 μm particles after applying the acoustic field at the channel’s end. **(E)** Graph of comparison color intensity across the microchannel before and after applying the SSAW field. (The lower color intensity corresponds to darker color).

**FIGURE 11:**
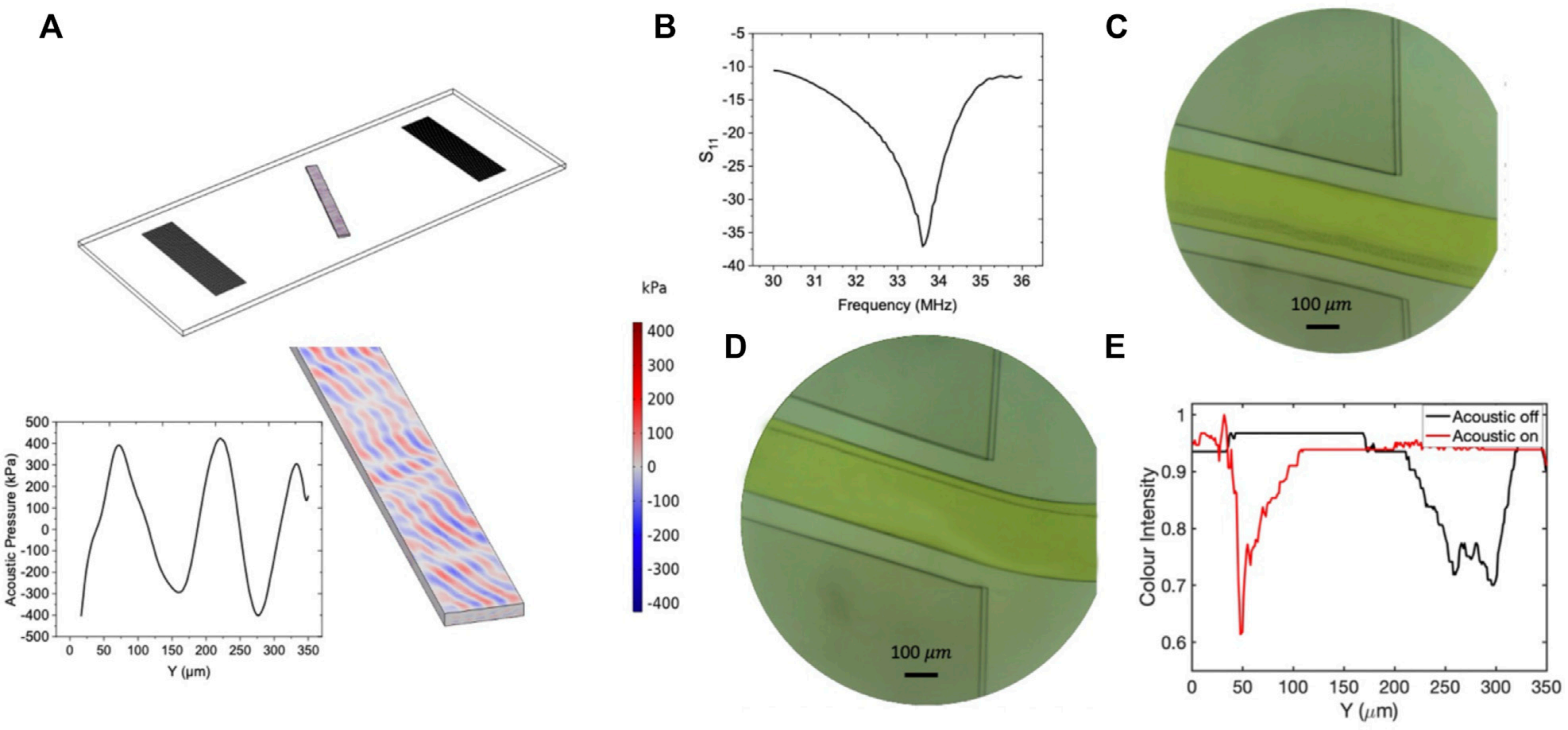
The results of the numerical and experimental studies corresponding to experiment number 90 with tilt angle 20°. **(A)** Contours of the acoustic pressure field distribution inside the fluidic domain. **(B)** The frequency response of the resonator. **(C)** The flow of 0.5 μm particles before applying the acoustic force at the channel’s end. **(D)** Confinement of 0.5 μm particles after applying the acoustic field at the channel’s end. **(E)** Graph of comparison color intensity across the microchannel before and after applying the SSAW field. (The lower color intensity corresponds to darker color).

The range of reflection coefficient for different geometrical configurations was observed between −9(dB) to −50 (dB) . To investigate the effect of reflection coefficient on particle confinement, two DOE suggested experiments with reflection coefficient values −14 (dB)  and −37 (dB) were selected for experimental and numerical studies. Figures 10, 11 demonstrate the acoustic pressure field and particle migration results for experiment numbers 36 and 90 with corresponding reflection coefficient values as −14 (dB) and −37 (dB)  , respectively. In these experiments, the applied voltage was considered 20 V, and the particle and sheath stream flowrates were as 3 and 5 μl/min . To avoid contacting the particles to channel wall, another sheath flow with 0.5 μl/min flowrate was flowed to impede the sticking of the particles to the channel wall. The three-dimensional simulation was carried out to investigate the pattern and magnitude of the acoustic radiation force. It was observed that there is a considerable difference in the acoustic pressure amplitude for these two devices. As Figures 10A, 11A demonstrate, the peak of the acoustic radiation force across the microchannel cross-section for the device with $S_{11}$ = −37 (dB) is 400 kPa , approximately double the device with $S_{11}=\;-14\;\left(dB\right)$ ( 200 kPa ). The pressure distribution contours (Figures 10A, 11A) reveal that for the device with a reflection coefficient value as −37 (dB) the acoustic pressure lines are aligned with 20° with respect to microchannel cross-section, while the contours of the pressure distribution for SAW device with a reflection coefficient of  −14 (dB) are parallel to the channel wall. The separation resolution strongly depends on the pattern of the acoustic pressure lines alignment. The highest focusing efficiency acquires when the acoustic pressure nodal lines are not parallel to channel walls and preserve their corresponding tilt angle with respect to microchannel cross-section. In case pressure nodal planes in three dimensions are parallel to the channel walls, the particle’s lateral migration will just be bounded to small movement to the closest nodal plane; however, for the separation purposes, the particles need to have complete lateral movement across the channel width, and this issue is only possible when the pressure nodes are formed tilted with respect to the mainstream flow direction. Figures 10C,D demonstrate the microbeads focusing for experiment number 36 with an absolute reflection coefficient value of 14 at the end of the microchannel before and after RF excitation at the corresponding resonant frequency (Figure 10B). The image processing technique based on the color intensity evaluation was employed to track the particle confinement across the microchannel width. The lower number in the vertical axis of Figure 10E shows the darker color or higher concentration of particles. As shown in Figure 10D, exposing SSAW to the particles does not contribute to the lateral movement of particles and only reduces the thickness of the particle flow path. This issue arises from the low capability of the chip in the transmission of the applied power to the particles and failure in maintaining the acoustic pressure tilt angle between the microchannel and IDTs. In this situation, the drag-induced force from the sheath flow is dominant over the acoustic radiation force, resulting in reducing focusing efficiency. As Figure 10E elucidates, before the SSAW excitation, the particles are focused within a span of 250 μm to 330 μm in the lateral direction (Y-axis). By applying the RF voltage, the SSAW-induced flow thickness was shrunk to 20 μm within a span of 300 μm (Figure 10E). The same study conducted for experiment number 90 with an absolute reflection coefficient value of 37 demonstrated in Figure 11. As Figure 11A demonstrates, the acoustic pressure nodal planes preserved their slanted formation by scattering inside the microchannel. The experimental photos before and after SAW exposure to the particles are elucidated in Figures 11C,D, respectively. Before applying the RF voltage, the side sheath flows focused on the particles within the span of 250 μm to 300 μm of the *Y*-axis. After acoustic field excitation, the particles moved to the upper side of the channel within the span of 45 μm to 55 μm of the Y axis, showing an excellent capability of the device in transmitting the input power to the particles. Further studies unfolded that for submicron particle separation purposes, the designed acoustic actuator must have a reflection coefficient below −30 (dB). It was observed that the devices with a reflection coefficient higher than −30 (dB) impose a small amount of force on submicron particles and cannot push them above the channel’s centerline. The alternative way to address this issue is increasing the applied power; however, increasing power contributes to cavitation, electrophoresis effect, and heat generation.

#### 3.4.2 Quality Factor

The other key parameter strongly affecting the isolation efficiency is the bandwidth. Finding the exact value of the resonant frequency requires the VNA machine, which is very expensive and space-consuming. Moreover, sometimes the resonant frequency does not stay constant and shifts slightly during the experiment. For devices with narrow bandwidth, the minute shift in the resonant frequency causes a remarkable rise in return loss, while the wideband coverage means that the system can still operate with some degree of deviation from the resonant frequency without any considerable change in return loss. The elimination of using VNA machine for finding the resonant frequency and benefiting from simple equation v=λf , (where v is the speed of sound in lithium niobate substrate, λ is the wavelength of the SAW resonator and f is the resonant frequency) can extensively reduce the cost and complexity of the experimental setup. Although this equation is inaccurate and does not give the exact resonant frequency, it can estimate the resonant frequency range. For narrow bandwidth operating resonators, the slight deviation from resonant frequency results in a drastic drop in power transmission; thereby, it is critical to avoid designing the devices with narrow bandwidth to accomplish efficient energy transmission. Multiple parameters influence the quality factor, piezoelectric dielectric loss, loading effects, ohmic losses, and leakage of the acoustic wave to the substrate.

**FIGURE 12:**
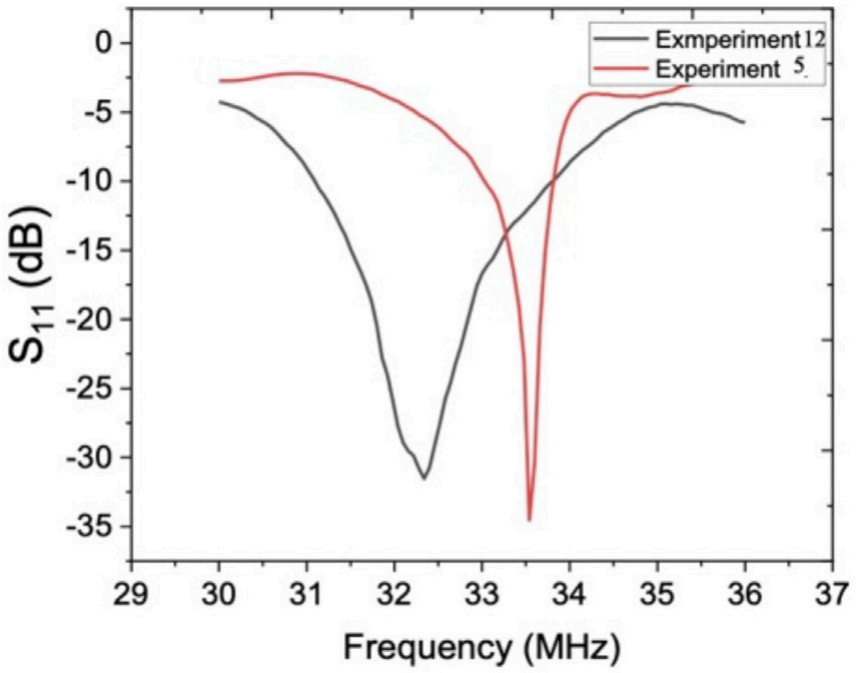
Frequency response of experiment numbers 12 and 5.

**FIGURE 13:**
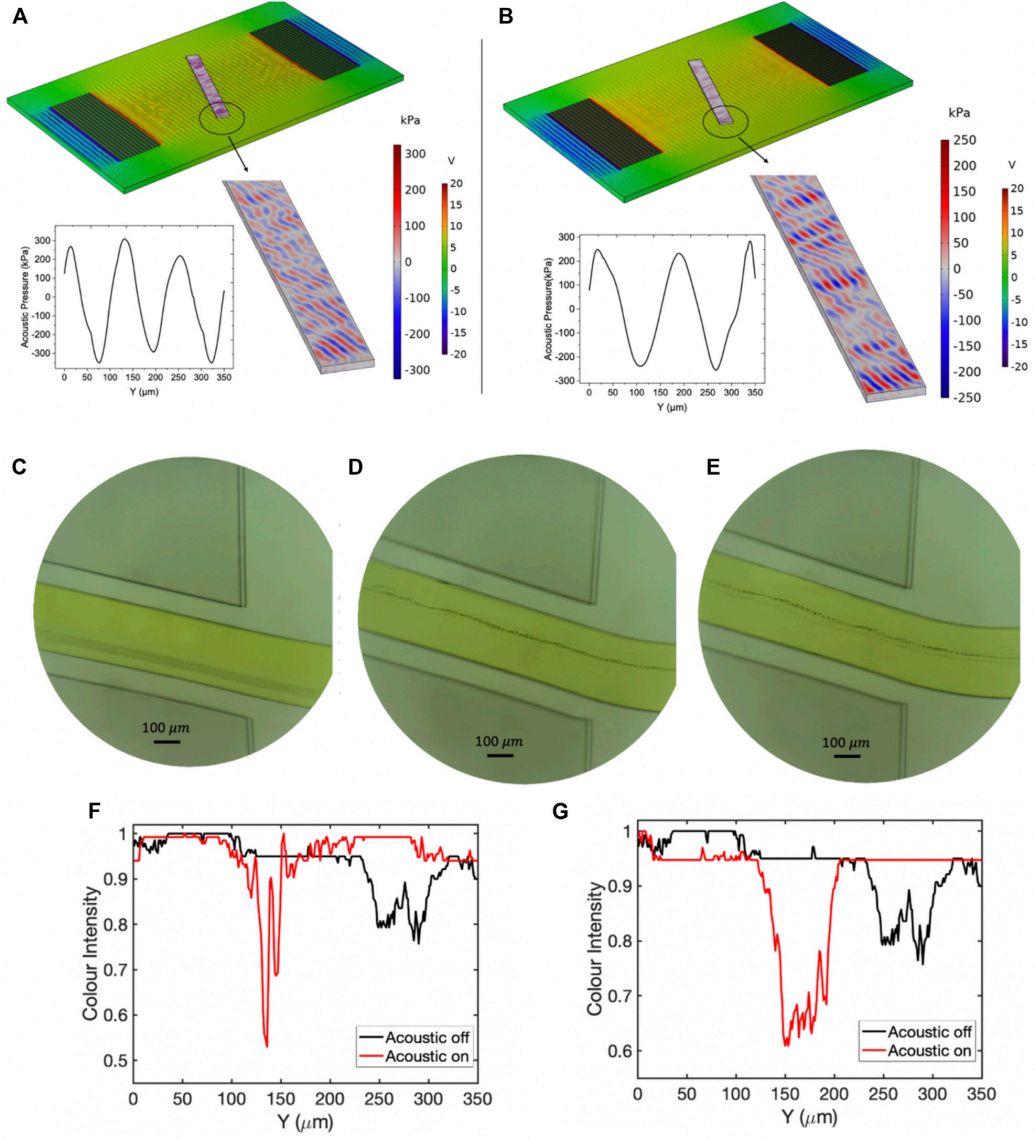
The results of pressure distribution and experimental tests for experiment number 12 with Q-factor 14 in resonant frequency and 0.5 MHz deviation from resonant frequency. **(A)** The distribution of pressure and voltage across the microchannel in resonant frequency. **(B)** The distribution of pressure and voltage across the microchannel with 0.5 MHz deviations from resonant frequency. **(C)** Sheath flow focusing of the stream of 0.5 μm particles before SSAW excitation. **(D)** Microbead focusing flow path after SSAW excitation in resonant frequency. **(E)** Particle flow path after SSAW excitation in 0.5 MHz off-resonant frequency. **(F)** Graph of colour intensity before and after acoustic actuation in resonant frequency **(G)** Graph of colour intensity before and after acoustic actuation in off-resonant frequency.

**FIGURE 14:**
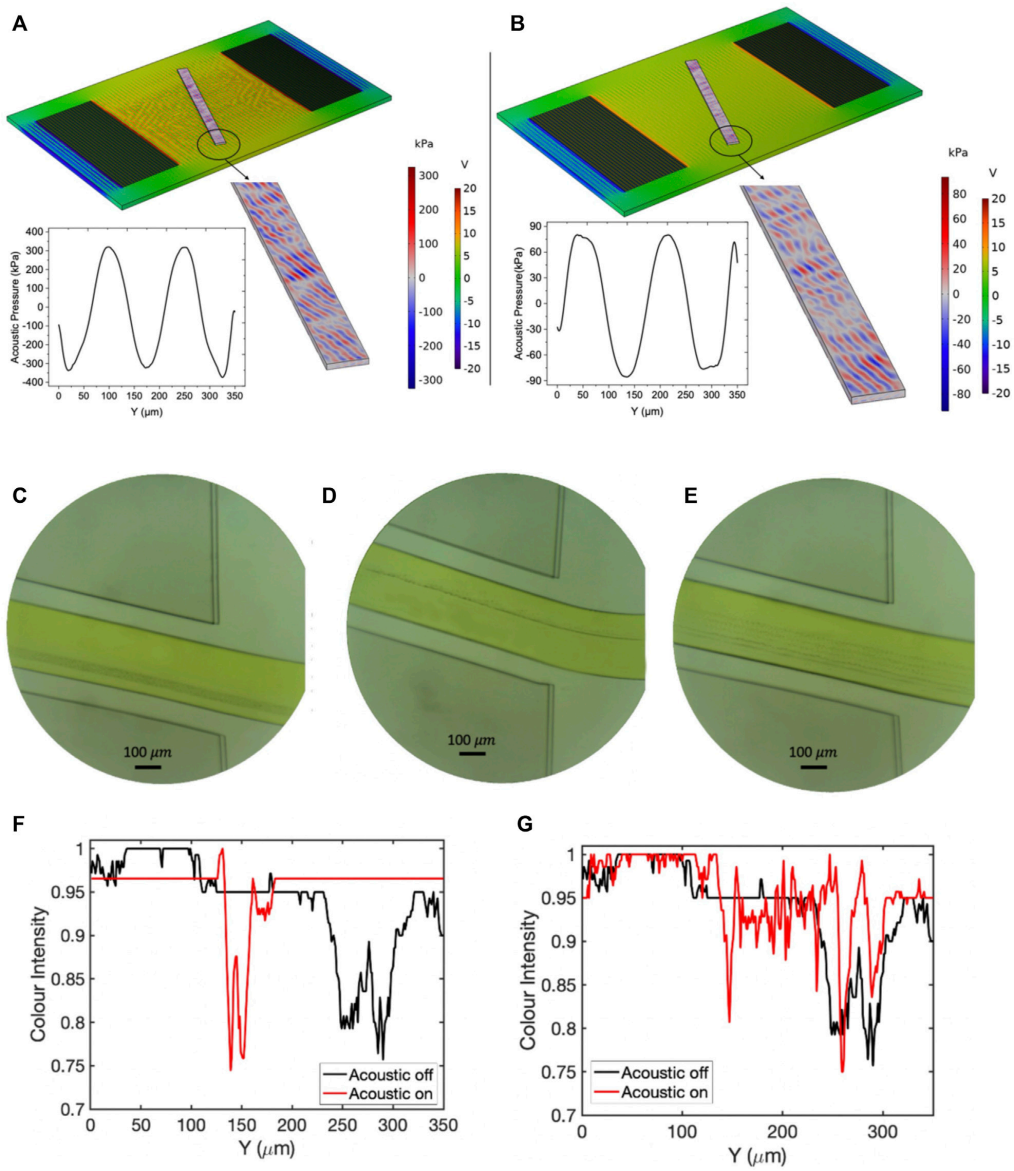
The results of pressure distribution and experimental tests for experiment number 5 with Q-factor 78 in resonant frequency and 0.5 MHz  deviation from resonant frequency. **(A)** The distribution of pressure and voltage across the microchannel in resonant frequency. **(B)** The distribution of pressure and voltage across the microchannel with 0.5 MHz deviations from resonant frequency. **(C)** Sheath flow focusing of the stream of 0.5 μm particles before SSAW excitation. **(D)** Microbead focusing flow path after SSAW excitation in resonant frequency. **(E)** Particle flow path after SSAW excitation in 0.5 MHz off-resonant frequency. **(F)** Graph of colour intensity before and after acoustic actuation in resonant frequency **(G)** Graph of colour intensity before and after acoustic actuation in off-resonant frequency.

To investigate the role of bandwidth on separation efficiency, we conducted two experiments with high and low-quality factors. Experiment number 12 with quality factor 14 and experiment number 5 with quality number 78 were selected for numerical and experimental studies. The frequency response of these two experiments are represented in Figure 12. As Figure 12 reveals, both devices show similar reflection coefficient value about −32 (dB) in their resonant frequency. Although the wavelength for both designs was the same, there was about 1 MHz difference in their resonant frequency, showing the role of geometrical features of the IDTs on the resonant frequency. However, due to the acoustic actuator’s extreme nonlinear behavior, linking the acoustic actuator parameters to the resonant frequency is not a viable route. The resonant frequency for experiment number 12 is 32.5 MHz and for experiment number 5 is 33.5 MHz . As Figure 12 indicates, 0.5 MHz rise in the resonant frequency for experiment number 5 increases the reflection coefficient value to −28 (dB),  while for experiment number 12, the 0.5 MHz rise in resonant frequency results in reflection coefficient as −10 (dB). The −10 (dB) return loss shows that a large portion of the applied power is reflected to the source and has not been transferred to the fluidic domain. The experimental and numerical studies were conducted to better demonstrate the bandwidth effect on separation efficiency. Figure 13 depicts the pressure distribution results and experimental tests for experiment number 12 with Q-factor 14 in resonant frequency and 0.5 MHz off from resonant-frequency. The exact value of the resonant frequency was measured by the VNA machine. Figure 13A demonstrates the contours of the pressure and voltage in resonant frequency transducer actuation, and Figure 13B depicts the same contours for 0.5 MHz off-frequency wave excitation. As it could be seen, the peak values of the acoustic pressure have remained almost constant for both resonant and off-resonant frequency actuation. The same behavior was also observed for the contours of voltage distribution in the piezoelectric and liquid domains. Figure 13C illustrates the sheath flow assisted focusing of 0.5 μm microbeads before applying the RF voltage. Figures 13D,E demonstrate the microbeads flow path in the resonant frequency and off-resonant frequency SSAW actuation at the end of the microchannel, respectively. As Figure 13f shows, before applying the RF voltage, the particles were focused on the span of 230 μm to 300 μm distance from the upper side of the channel in the Y-axis. By applying the RF voltage in resonant frequency, the particles focusing range was changed to 130 μm to 150 μm . By slightly shifting the excitation frequency from measured resonant frequency to 0.5 MHz off-resonant frequency, it was observed that the focusing range of the particles was 20 μm wider compared to resonant frequency actuation and was focused on the span of 150 μm to 200 μm distance from the upper side of the channel at the end of the microchannel (Figure 13G). The comparison of Figures 13D,E shows that all the particles were not focused on a single line for off-resonant actuation, unlike the resonant actuation where all the particles were focused in one flow stream. As Figure 13E depicts, the particles are focused in two parallel lines while the upper line contains the higher concentration of microbeads. This parallel formation of particles flow stream is due to the slight drop in power transmission due to return losses from off-resonant frequency excitation. The same study was carried out for experiment number 5 with a Q-factor value of 78. The contours of pressure distribution and experimental studies are depicted in Figure 14. The high value of the Q-factor for experiment number 5 shows that the slight deviation from resonant frequency can result in a remarkable drop in power transmission to the particles. Figures 14A,B represent the contours of the voltage and pressure distribution for resonant frequency actuation and 0.5 MHz off-resonant frequency actuation. As seen, the acoustic pressure’s peak values are declined from 300 kPa for resonant actuation to 80 kPa for off-resonant frequency actuation (73% drop in acoustic pressure). It was also seen that the nodal pressure lines could not preserve their tilted formation for off-resonant actuation in the channel, and nodal planes were formed parallel to the channel walls, which is not favorable for separation purposes. Comparing the contours of voltage for these two experiments shows that in resonant frequency with 20 V  initial excitation, about 17 V is delivered to the piezoelectric underneath the microchannel, while this value for the off-resonant actuation was about 5 V . Figures 14C,D,E show the flow path of the microbeads, before acoustic excitation, resonant frequency actuation, and 0.5 MHz  off-resonant actuation for experiment number 5. As Figures 14F,G represent, before applying the acoustic field the particles are focused in the span of 250 μm to 300 μm from the upper side of the microchannel. By actuation of the transducer in resonant frequency, the particles were moved to the upper side of the microchannel and focused on the span of 140 μm to 155 μm from the upper microchannel wall. As Figure 14E demonstrates, the non-tilted formation of nodal planes has led to particles focusing on multiple parallel lines. Due to power reflection from the transducer, the power is not fully delivered to the particles. The acoustic radiation force acting on the particles was insufficient to overcome the drag-induced force from sheath-flow.

## 4 Conclusion

A learning-based approach for optimizing an acoustic transducer by combining the ANN and MOGA was introduced for the first time. The developed ANNs revealed an equivalent accuracy to experimental test with $R^{2}=0.97$ for reflection coefficient and $R^{2}=0.96$ for quality factor with very low computation time. For multi-objective optimization, NSGA-II is utilized as a MOGA tool to meet piezotransducer optimization requirements and provide a valid set of solutions according to the combination of suitable variables. The ANN-GA model was successfully used as a design guideline for suggesting the optimum IDTs structural configurations of the SAW resonator by establishing a Pareto solution set of optimal reflection coefficient and quality factor. Due to the IDT design’s critical role on the SAW device’s frequency response, the design parameters were considered as the geometrical variables of the IDTs (i.e., electrode length, distance between electrodes, and number of fingers). The finite element methodology coupled with experimental studies were used for the full-scale analysis (acoustic pressure amplitude, distribution, voltage, particle migration and thermal effect) of the piezotransducer. To minimize the sound wave attenuation inside the PDMS layer, 100 μm acoustic window was designed to reduce the power loss inside the PDMS domain. By conducting the sensitivity analysis on the reflection coefficient and quality factor, it was observed that the reflection coefficient and Q-factor significantly influence the acoustic pressure amplitude and distribution. It was observed that increasing RF signal voltage excitation above $24V_{pp}$ increases the risk of bubble formation in the microchannel due to the cavitation. Apart from finding the optimal set of solutions by combined ANN-MOGA, this technique can also be extended to creating a framework for suggesting the design parameters for user-specified performance needs. Chen and Wang, (2021).

## Data Availability

The raw data supporting the conclusion of this article will be made available by the authors, without undue reservation.
