# Supplementary material for: Neural Network-Based Optimization of an Acousto Microfluidic System for Submicron Bioparticle Separation
Source: Front Bioeng Biotechnol. 2022 Apr 19;10:878398. doi: 10.3389/fbioe.2022.878398 (PMC9061962; doi:10.3389/fbioe.2022.878398)
Supplement: Supplementary file 3 [file DataSheet1.docx]

## **Impedance matching**

All the electroacoustic devices can be considered as a network with multiple ports, and each port can transmit, reflect or absorb the radiofrequency energy. The scattering parameters (S-parameters) are the most common ways of representing the network energy response. In most cases, the network shows the frequency-specific behavior so that scattering parameters are measured over the frequency range. The geometrical configuration of IDTs is the most critical factor in defining the reflection coefficient ${(S}_{11})$. The IDTs with a high value of reflection coefficient reflect most of the radiofrequency energy so that acoustic energy will not be enough to deviate the particles for separation purposes.

The performance analysis of the acoustofluidic device can be evaluated by two main factors, the return power loss and the system's bandwidth. The periodic array of electrodes, piezoelectric, and microchannel, can be considered a single network with two ports, and the performance of the whole device could be done through network analysis. The main factor in characterizing the circuit performance are scattering parameters or S-parameters. The S-parameters define the relationship between the network's incident and reflected power waves. To achieve the highest power transfer and minimum reflection, it is essential to match the impedance of the driving source, transmission line, and acoustic actuator. As the function generator and amplifier have an internal impedance of $50 \Omega$, the acoustic actuator should also have a $50 \Omega$ impedance to reach a fully matched system. The assessment of impedance matching quality is performed through the complex reflection coefficient (Γ) parameter. The reflection coefficient definition is represented in Eq. 1 where $Z_{L}$ is the load impedance and $Z_{S}$ is the source impedance.

| $\Gamma=\rho\theta=\frac{Z_{L}-Z_{S}}{Z_{L}+Z_{S}}$ | Eq. 1 |
| --- | --- |

The perfect matching occurs when the load and source impedance are equal ($Z_{L}=Z_{S})$, leading to zero reflection coefficient, meaning that all the power is delivered to the load. In case the load impedance is infinite (i.e., an open circuit) or load impedance is zero (i.e., a short circuit), the magnitude of Γ is one showing the complete reflection of the incident wave. The input reflection coefficient ($S_{11}$ expressed in dB), which expresses the amount of the power not absorbed by the acoustic actuator and returned to the source, can be calculated by Eq. 2.

| $S_{11}(dB)=-20log(\left\vert\Gamma\right\vert)$ | Eq. 2 |
| --- | --- |

The lower reflection coefficient value implies a better match and power transmission. To design a fully matched system, first we need to find the values of the equivalent circuit model and equal the real part of the circuit impedance as $50 \Omega$. The equivalent circuit model of the SAW resonator can be expressed as Modified Butterworth-Van Dyke (MBVD) model shown in Fig. 2.


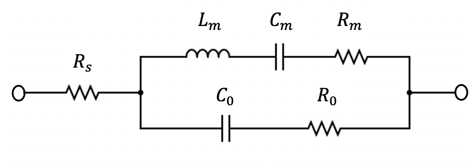


Fig. 2 Modified Butterworth-Van Dyke (MBVD) acoustic resonator equivalent circuit model

The uppper branch of the model with subscript $m$ corresponds to the mechanical response and the lower branch includes the electrical response of the SAW actuator. In Fig. 2, the terms $R_{m}$ is the motional resistance, $C_{m}$ is the motional capacitance,$L_{m}$ is the motional inductance, $C_{0}$ is the static capacitance formed by the piezoelectric between electrodes, $R_{0}$ is the dielectric loss and $R_{s}$ is the resitance of the electrodes[1]. The analytical expression of the total impedance of the MBVD model has been expressed in Eq. 3.

| $Z\left( \omega\right)=R_{s}+\frac{(R_{0}+\frac{1}{j\omega C_{0}})(R_{m}++\frac{1}{j\omega C_{m}}+j\omega L_{m})}{R_{0}+\frac{1}{j\omega C_{0}}+R_{m}+\frac{1}{j\omega C_{m}}+j\omega L_{m}}$ | Eq. 3 |
| --- | --- |

As it could be seen, the impedance calculation of the SAW resonator is highly complex and include multiple components that need to be derived. To address the mismatching problem, it is inevitable to use matching circuits to increase the energy conversion between the source and SAW resonator. However, a matching network is an extra unit added to the system and increases the cost and complexity of the setup. Moreover, finding the proper values and components of the matching network is not straightforward and should be changed with any changes in the IDT design. In some cases, the unsuitable matching network design shows good matching by increasing the power loss in the network rather than improving the SAW excitation. Taking all these together, designing the SAW actuator with $50 \Omega$ impedance is critical to eliminate adding an extra matching circuit to the system. Another important parameter describing the performance of the acoustic wave resonator is the quality factor (Q-factor) defined as $Q=f_{r}/\Delta f$, where $f_{r}$ is the resonant frequency and $\Delta f$ is the frequency difference corresponding to one-half of the peak’s highest amplitude. The Q-factor in the RF oscillators is a measure of the bandwidth assessment of the resonant circuit. The smaller value of the Q-factor corresponds to the broader bandwidth coverage. The wide bandwidth operational criterion should be considered in resonator design to reduce the resonance selectivity.

Table S1. The physical properties of different materials used for the numerical simulation.

| Material | Symbol | Value |
| --- | --- | --- |
| **Water** |  |  |
| Density | $\rho_{0}$ | 997 $[kg/m^{3}$] |
| Speed of sound | $c_{0}$ | 1497 $[m/s]$ |
| Shear viscosity | $\mu$ | $890 [Pa.s]$ |
| Bulk viscosity | $\mu_{b}$ | $2.47 [mPa.s]$ |
|  |  |  |
| **PDMS** |  |  |
| Density | $\rho_{PDMS}$ | 920 $[kg/m^{3}$] |
| Speed of sound | $c_{PDMS}$ | 1076.5 $[m/s]$ |
| Attenuation coefficient @ 6.65 $MHz$ | $\alpha_{PDMS}$ | $31 [dB/cm]$ |
|  |  |  |
| **Polystyrene particle** |  |  |
| Density | $\rho_{PS}$ | 1060 $[kg/m^{3}$] |
| Speed of sound | $c_{PS}$ | 2050 $[m/s]$ |

Table S2. Results of the conducted experiments for full factorial design of experiment.

| Experiment number | | $L_{1}(mm)$ | | $L_{2}(mm)$ | | Number of fingers (NF) |  |
| --- | --- | --- | --- | --- | --- | --- | --- |
| 1 | | 6 | | 5 | | 34 |  |
| 2 | | 6 | | 8 | | 20 |  |
| 3 | | 10 | | 8 | | 24 |  |
| 4 | | 8 | | 4 | | 34 |  |
| 5 | | 9 | | 5 | | 40 |  |
| 6 | | 10 | | 7 | | 40 |  |
| 7 | | 7 | | 5 | | 24 |  |
| 8 | | 9 | | 6 | | 20 |  |
| 9 | | 8 | | 7 | | 30 |  |
| 10 | | 7 | | 5 | | 30 |  |
| 11 | | 9 | | 4 | | 34 |  |
| 12 | | 6 | | 5 | | 24 |  |
| 13 | | 6 | | 7 | | 34 |  |
| 14 | | 6 | | 4 | | 30 |  |
| 15 | | 7 | | 8 | | 30 |  |
| 16 | | 6 | | 4 | | 24 |  |
| 17 | | 9 | | 6 | | 40 |  |
| 18 | | 9 | | 6 | | 34 |  |
| 19 | | 6 | | 6 | | 20 |  |
| 20 | | 8 | | 6 | | 24 |  |
| 21 | | 9 | | 4 | | 24 |  |
| 22 | | 7 | | 5 | | 40 |  |
| 23 | | 6 | | 7 | | 40 |  |
| 24 | | 7 | | 4 | | 30 |  |
| 25 | | 8 | | 4 | | 30 |  |
| 26 | | 8 | | 8 | | 30 |  |
| 27 | | 10 | | 6 | | 40 |  |
| 28 | | 10 | | 8 | | 40 |  |
| 29 | | 10 | | 4 | | 30 |  |
| 30 | | 10 | | 6 | | 30 |  |
| 31 | | 8 | | 5 | | 34 |  |
| 32 | | 6 | | 6 | | 40 |  |
| 33 | | 7 | | 5 | | 20 |  |
| 34 | | 9 | | 6 | | 24 |  |
| 35 | | 8 | | 7 | | 40 |  |
| 36 | | 6 | | 8 | | 40 |  |
| 37 | | 7 | | 6 | | 30 |  |
| 38 | | 9 | | 5 | | 34 |  |
| 39 | | 6 | | 8 | | 34 |  |
| 40 | | 10 | | 7 | | 24 |  |
| 41 | | 8 | | 6 | | 20 |  |
| 42 | | 9 | | 5 | | 30 |  |
| 43 | | 8 | | 6 | | 40 |  |
| 44 | | 6 | | 6 | | 30 |  |
| 45 | | 6 | | 7 | | 30 |  |
| 46 | | 6 | | 5 | | 40 |  |
| 47 | | 10 | | 6 | | 34 |  |
| 48 | | 9 | | 4 | | 30 |  |
| 49 | | 10 | | 7 | | 34 |  |
| 50 | | 8 | | 4 | | 40 |  |
| 51 | | 9 | | 8 | | 20 |  |
| 52 | | 10 | | 4 | | 40 |  |
| 53 | | 7 | | 7 | | 34 |  |
| 54 | | 10 | | 5 | | 20 |  |
| 55 | | 7 | | 8 | | 24 |  |
| 56 | | 10 | | 8 | | 34 |  |
| 57 | | 8 | | 7 | | 34 |  |
| 58 | | 10 | | 4 | | 24 |  |
| 59 | | 8 | | 5 | | 40 |  |
| 60 | | 8 | | 8 | | 34 |  |
| 61 | | 10 | | 4 | | 20 |  |
| 62 | | 8 | | 5 | | 24 |  |
| 63 | | 8 | | 8 | | 20 |  |
| 64 | | 6 | | 8 | | 24 |  |
| 65 | | 7 | | 7 | | 24 |  |
| 66 | | 8 | | 4 | | 24 |  |
| 67 | | 9 | | 7 | | 24 |  |
| 68 | | 10 | | 7 | | 30 |  |
| 69 | | 8 | | 5 | | 30 |  |
| 70 | | 9 | | 8 | | 24 |  |
| 71 | | 7 | | 8 | | 34 |  |
| 72 | | 7 | | 4 | | 40 |  |
| 73 | | 7 | | 7 | | 20 |  |
| 74 | | 7 | | 6 | | 34 |  |
| 75 | | 6 | | 4 | | 34 |  |
| 76 | | 9 | | 6 | | 30 |  |
| 77 | | 6 | | 5 | | 30 |  |
| 78 | | 6 | | 8 | | 30 |  |
| 79 | | 9 | | 8 | | 30 |  |
| 80 | | 6 | | 6 | | 34 |  |
| 81 | | 7 | | 7 | | 30 |  |
| 82 | | 7 | | 4 | | 20 |  |
| 83 | | 9 | | 7 | | 34 |  |
| 84 | | 9 | | 7 | | 20 |  |
| 85 | | 8 | | 4 | | 20 |  |
| 86 | | 6 | | 7 | | 24 |  |
| 87 | | 10 | | 6 | | 20 |  |
| 88 | | 9 | | 7 | | 30 |  |
| 89 | | 7 | | 8 | | 40 |  |
| 90 | | 7 | | 8 | | 20 |  |
| 91 | | 9 | | 8 | | 40 |  |
| 92 | | 6 | | 4 | | 20 |  |
| 93 | | 8 | | 8 | | 40 |  |
| 94 | | 8 | | 7 | | 20 |  |
| 95 | | 9 | | 5 | | 20 |  |
| 96 | | 6 | | 5 | | 20 |  |
| 97 | | 10 | | 5 | | 40 |  |
| 98 | | 8 | | 6 | | 30 |  |
| 99 | | 7 | | 6 | | 20 |  |
| 100 | | 8 | | 5 | | 20 |  |
| 101 | | 9 | | 7 | | 40 |  |
| 102 | | 10 | | 5 | | 24 |  |
| 103 | | 6 | | 6 | | 24 |  |
| 104 | | 10 | | 5 | | 30 |  |
| 105 | | 8 | | 8 | | 24 |  |
| 106 | | 7 | | 6 | | 24 |  |
| 107 | | 9 | | 5 | | 24 |  |
| 108 | | 9 | | 4 | | 40 |  |
| 109 | | 7 | | 5 | | 34 |  |
| 110 | | 8 | | 7 | | 24 |  |
| 111 | | 7 | | 4 | | 34 |  |
| 112 | | 10 | | 5 | | 34 |  |
| 113 | | 7 | | 6 | | 40 |  |
| 114 | | 8 | | 6 | | 34 |  |
| 115 | | 6 | | 7 | | 20 |  |
| 116 | | 7 | | 7 | | 40 |  |
| 117 | | 10 | | 7 | | 20 |  |
| 118 | 6 | | 4 | | 40 | | |
| 119 | 10 | | 4 | | 34 | | |
| 120 | 10 | | 6 | | 24 | | |
| 121 | 7 | | 4 | | 24 | | |
| 122 | 9 | | 4 | | 20 | | |
| 123 | 10 | | 8 | | 20 | | |
| 124 | 10 | | 8 | | 30 | | |
| 125 | 9 | | 8 | | 34 | | |

[1] L. Chen and Y. Wang, “Dependence of modified butterworth van-dyke model parameters and magnetoimpedance on dc magnetic field for magnetoelectric composites,” *Materials (Basel).*, vol. 14, no. 16, 2021, doi: 10.3390/ma14164730.
